# Median Effective Analgesic Concentration of Ropivacaine in Ultrasound-Guided Interscalene Brachial Plexus Block as a Postoperative Analgesia for Proximal Humerus Fracture: A Prospective Double-Blind Up-Down Concentration-Finding Study

**DOI:** 10.3389/fmed.2022.857427

**Published:** 2022-05-06

**Authors:** Yang Liu, Cheng Xu, Chengyu Wang, Fei Gu, Rui Chen, Jie Lu

**Affiliations:** ^1^Department of Anaesthesiology, Shanghai Jiaotong University Affiliated Sixth People's Hospital, Shanghai, China; ^2^Department of Anaesthesiology, Hainan Hospital of GLA General Hospital, Shanghai, China

**Keywords:** interscalene brachial plexus block, median effective analgesic concentration, postoperative analgesia, proximal humerus fracture, Ropivacaine

## Abstract

**Background:**

The innervation of the proximal humerus fracture is complicated and unclear. The use of interscalene nerve block has been effective as postoperative analgesia for patients, but the optimal concentration of usage is unknown.

**Method:**

This study was conducted on 30 patients with ASA I or II, who were planning to undergo a proximal humerus fracture operation. A dosage of 10 ml Ropivacaine was administered for the interscalene brachial plexus block (ISBPB) as determined using the up-and-down sequential method. The initial concentration of Ropivacaine in the first patient to receive ISBPB was 0.3%. After a successful or unsuccessful postoperative analgesia, the concentration of local anesthetic was decreased or increased, respectively, by 0.05% in the next patient. We defined successful postoperative analgesia as a visual analog scale (VAS) score of < 4 at rest, within the initial 8 h after ISBPB. The analytic techniques of linear, linear-logarithmic, exponential regressions, and centered isotonic regression were used to determine the EC50 of Ropivacaine, and the residual standard errors were calculated for the comparison of “goodness of fit.”

**Results:**

The concentration of Ropivacaine ranged from 0.1 to 0.35%. The EC50 (95% confidence interval) from 4 different statistical approaches (linear, linear-logarithmic, exponential regressions, and centered isotonic regression) were 0.222% (0.198%, 0.335%), 0.233% (0.215%, 0.453%), 0.223% (0.202%, 0.436%), and 0.232%, respectively. Among all the 4 models, the linear regression had the least residual standard error (0.1676).

**Conclusion:**

The EC50 from the four statistical models for 10 ml Ropivacaine in ultrasound-guided ISBPB for postoperative analgesia was distributed in a narrow range of 0.222–0.233%.

**Trial Registration:**

www.chictr.org.cn/; registration number: ChiCTR2100047231.

## Introduction

Proximal humeral fractures are common and may account for up to 10% of all fractures in the elderly population over 60 years, with a notably higher incidence in women aged 80 to 89 years (1, 2). Whether to use conservative or surgical treatment mainly depends on the fracture pattern and the functional demands of the patient. At present, for complex, unstable, or severe proximal humeral fractures, surgical is the commonly accepted treatment (3, 4). However, this is often associated with significant pain, with patients often receiving multiple doses of opiate medications, which affects the quality of life and is related to high mortality rates (5).

Traditional proximal humeral surgery generally uses “beach chair position” or “semi-sitting position.” To better manage the airway and provide patients with more comfort, general anesthesia is used. This study showed that general anesthesia, combined with interscalene brachial plexus block (ISBPB), could reduce the use of intraoperative opioid drugs, shorten postoperative recovery time, and alleviate postoperative pain (6). For ISBPB, some studies have shown that high concentrations of local anesthetics can increase the incidence of phrenic nerve paralysis and affect respiratory function (7). Therefore, determination of the median effective analgesic concentration (MEAC, EC50 = effective concentration in 50% of patients) is important.

Ropivacaine is one of the commonly used analgesics for nerve block. It has the advantages of fast onset, long-acting time, fewer incidences reported of arrhythmia than bupivacaine, and rare severe central nervous system toxicity and cardiovascular toxicity (8). Studies have found that brachial plexus block with 0.1–0.3% Ropivacaine can achieve separation of sensory and motor, which provides the possibility of early postoperative functional exercise for patients (9).

This study aimed to estimate the MEAC of Ropivacaine used in ultrasound-guided ISBPB for successful postoperative analgesia of proximal humeral fractures.

## Methods

### Study Design and Population

This single-armed prospective study was approved by the Ethics Committee of the Sixth People's Hospital of Shanghai (reference No. 2021-144) and registered with the Clinical Trial Registry of China (http://www.chictr.org.cn/; registration No. ChiCTR2100047231; date of registration, June 11, 2021; date of patient enrollment, July 10, 2021). All patients who underwent proximal humerus fracture operation were assessed for eligibility. All eligible patients obtained written informed consent. Inclusion criteria: age between 18 and 70 years old, ASA physical status 1–2, and body mass index (bmi) between 18 and 35 kg/m^2^. Exclusion criteria: pregnancy, local infection at the block site, pre-existing neuropathy or coagulopathy, allergy to local anesthetics and opioids, dementia, known history of intravenous (IV) drug abuse, preoperative chronic opioid requirements, chronic pain, psychiatric illness, patients who failed to understand the scoring systems used in the study, uncontrolled hypertension or ischemic heart disease, renal or hepatic dysfunction, and pre-existing neurologic deficits.

### Blinding Method

All blocks were performed by one experienced anesthetist (G), using the same high-frequency (6 to 13 MHz) ultrasound probe (Sonosite, Inc., USA). Another anesthetist performed anesthesia management in the operating theater. An independent research assistant evaluated the nerve block. All personnel were blinded to the concentration of local anesthetic injected. A nurse, who did not participate in follow-up research, prepared the local anesthetics depending on the response of the previous patients.

### The Technique of Block Administration

Routine monitors (pulse oximeter, non-invasive blood pressure cuff, and electrocardiogram) were used, and intravenous access was established. Patients were positioned supine with the head turned 45 degrees to the non-operative side. After skin disinfection, the brachial plexus at the interscalene groove was identified either by distal-to-proximal (trace-back) approach or by medial-to-lateral approach. After clearly identifying root C5, C6, and C7 in the imaging screen, a 4-cm 22-gauge insulated needle (UniPlex Nanoline; Pajunk, Geisingen, Germany) was inserted using an in-plane technique from the lateral-to-medial direction. The needle tip was ultimately positioned close to each root at the 3 o'clock position, respectively. If paraesthesia was complained of, the needle tip was repositioned before local anesthetic (LA) injection to avoid nerve injury. A total of 10 ml (3–4 ml/root) of Ropivacaine was given, and the spread of local anesthetic was seen. All injections were administered slowly with a repeated aspiration to prevent or detect early intravascular injection. A concentration of 0.3% Ropivacaine was administered in the first patient. The Dixon and Mood's up-and-down study design was followed (10). LA concentration for subsequent patients was determined by success or failure of postoperative analgesia (success of postoperative analgesia: in the initial 8 h after ISBPB, the VAS score was < 4) in the previous patient. Drug concentration was increased by 0.05% in case of failure and decreased by 0.05% in case of success.

### Block Evaluation

Final needle removal time was noted as “block time”. Block assessment was done at 5-min intervals by an independent observer who was blinded to LA concentration until 30 min after block time. Sensory blockade was assessed on the deltoid and lateral upper arm according to a 3-point qualitative scale with a pinprick sensation test using a sharp 25 G needle: 0 = no block (compared with the contralateral side); 1 = incomplete block (a non-sharp sensation, touch or pressure); 2 = complete block (unable to recognize pinprick sensation). The motor block was assessed using a 3-point modified Bromage score: 0 = no motor block at full extension and flexion of all upper extremity joints; 1 = decreased motor strength with the ability to move only the fingers; 2 = complete motor block with the inability to move the elbow, wrist, and fingers.

### Clinical Procedures

General anesthesia was induced with propofol (1–2 mg/kg), sufentanil (0.1–0.15 μg/kg), and a laryngeal mask airway was placed at the proper position. Volatile anesthetics sevoflurane was used for maintenance, with end-expiratory sevoflurane concentration above 0.7 MAC (minimum alveolar concentration) and ETCO2 between 35 and 45 mmHg.The patient's spontaneous breathing was observed. During the operation, the anesthesiologist would use 0.1 μg/kg sufentanil intravenously if any signs indicated insufficient anesthesia (an increase of more than 20% in the heart rate and/or blood pressure compared to before anesthesia, rapid shallow breathing with a spontaneous respiratory rate greater than 20 breaths per minute). All patients received Postoperative nausea and vomiting (PONV) prophylaxis droperidol IV before emergence. When the surgical operation was completed, the patients were transferred to the post anesthesia care unit (PACU), and then, to the wards for discharge. For excluded patients, endotracheal intubation general anesthesia was performed. They were provided with a patient-controlled analgesia pump (sufentanil 1 ug/ml, background infusion 1ml/h, bolus 2 ug, and lockout 15 min) for 48 h postoperatively. Besides, an oral paracetamol 1 g or ibuprofen 400 mg could be given every 6 h after the surgery.

### Pain Assessment and Management

Patients were instructed to record their pain using the visual analog scale (VAS) (0–10, 0 = no pain, 10 = worst imaginable pain). VAS of rest pain and movement-related pain was measured immediately after resuscitation, right before discharging from the PACU, and at 4, 6, 8, and 24 h after the block time. The timing and dosage of analgesics were recorded. Twenty-four hours after the block time, patients were questioned for VAS, time of the first operative limb pain, and satisfaction with the ISBPB (0–3, 0 = very unsatisfied; 3 = very satisfied). In addition, patients were telephone-interviewed if they suffered a late complication such as nerve injury and pain radiating to the arm and forearm related to ISBPB after discharging from the hospital.

### UDM

A concentration of 0.3% of 10-ml Ropivacaine was administered in the first patient. After successful postoperative analgesia (in the initial 8 h after ISBPB, the VAS score was <4), the concentration of local anesthetic in the next patient was decreased by 0.05%. However, if the block was unsuccessful, then the local anesthetic concentration was increased by 0.05% in the next patient. All patients received < 3 mg/kg of Ropivacaine to avoid local anesthetic toxicity.

### Adverse Effect

Complications include hematoma, Horner's syndrome, hoarseness, nausea, vomiting, local anesthetic systemic toxicity (blurred vision, hearing impairment, sleep disturbances, dizziness, muscle twitching, and arrhythmia), respiratory distress, and hypoxemia, which were also assessed during this study.

### Statistical Analysis

In most cases, the exact sample size for Dixon's Up-and-down method (UDM) could not be determined in advance. When six cross-overs (conversion from successful block to unsuccessful block or vice versa) had occurred, we ceased to recruit patients (11). We determined that at least 20–40 patients would be required to provide reliable estimates of the target dose in our simulation studies in anesthesia trials using Dixon's UDM. Our study recruited 30 patients to achieve this goal.

To explore the target dose of EC50, four statistical approaches were used, including 3 parametric estimates of the dose-responsive curve (12): linear, linear-logarithmic and exponential regressions, and one nonparametric model: the centered isotonic regression, which was only for assuming a nondecreasing dose and response relationship (11).

The residual standard errors, a statistical tool to determine the goodness of fit, which analyzes how well a set of data points fit with the actual model, were calculated for all four statistical approaches. We also calculated Pearson's correlation coefficient (r) to find the association between the time to the first analgesic request and administered local anesthetic volume.

For the continuous variables, data were presented as mean ± SD or median (interquartile range) depending on the distribution of the data. For all categorical variables, frequency/percentage was calculated. The Mann–Whitney U test was used for statistical analysis of skewed continuous variables or ordered categorical data. Chi-square or Fisher exact test was applied to find out the association between subgroup and categorical variables.

## Results

**Figure 1:**
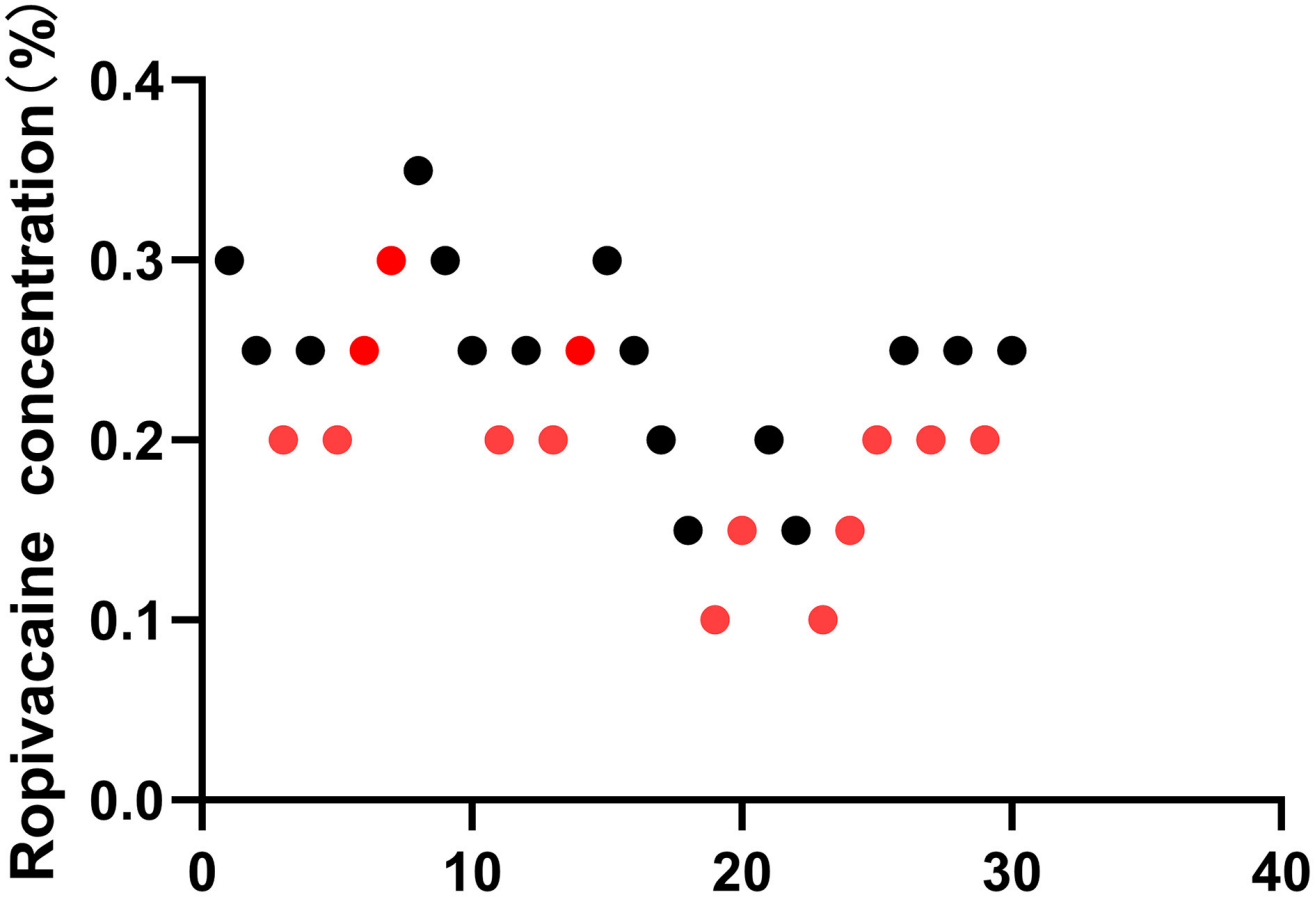
Sequential block results of ultrasound-guided Interscalene Brachial Plexus Block using 10 ml ropivacaine according to the Dixon and Massey up-and-down method.

All 30 patients in this study met the screening criteria, and no patients were excluded during the study. All patients were selected with eight independent up-down deflections (Figure 1). There was no significant difference in sex, age, BMI, ASA status, and duration of surgery between the upper and lower cases (*P* < 0.05). Table 1 shows the surgical characteristics of these patients.

**Table 1:** Patient characteristic.

**Characteristic**	**Mean ±SD or No. (%)**
Sex (male/female)	23/7
Age (yr)	36.2 ± 6.34
Body mass index (kg/m^2^)	22.7 ± 3.07
ASA physical status (I/II)	14/16
Duration of surgery (min)	67.9 ± 18.89
sufentanil consumption (μg)	8.3 ± 2.71
Time to 1st rescue analgesic (h)	7.4 ± 2.36
Time to remove the laryngeal mask (min)	9.8 ± 3.54
Onset time of sensory block (min)	5.0 ± 1.96
Onset time of motor block (min)	11.9 ± 2.73
Duration of motor block (h)	8.8 ± 2.20
Analgesic satisfaction (1/2/3)	0/10/20

*ASA, American Society of Anesthesiologists*.

### The Median Effective Analgesic Concentration of Local Anesthetic

**Figure 2:**
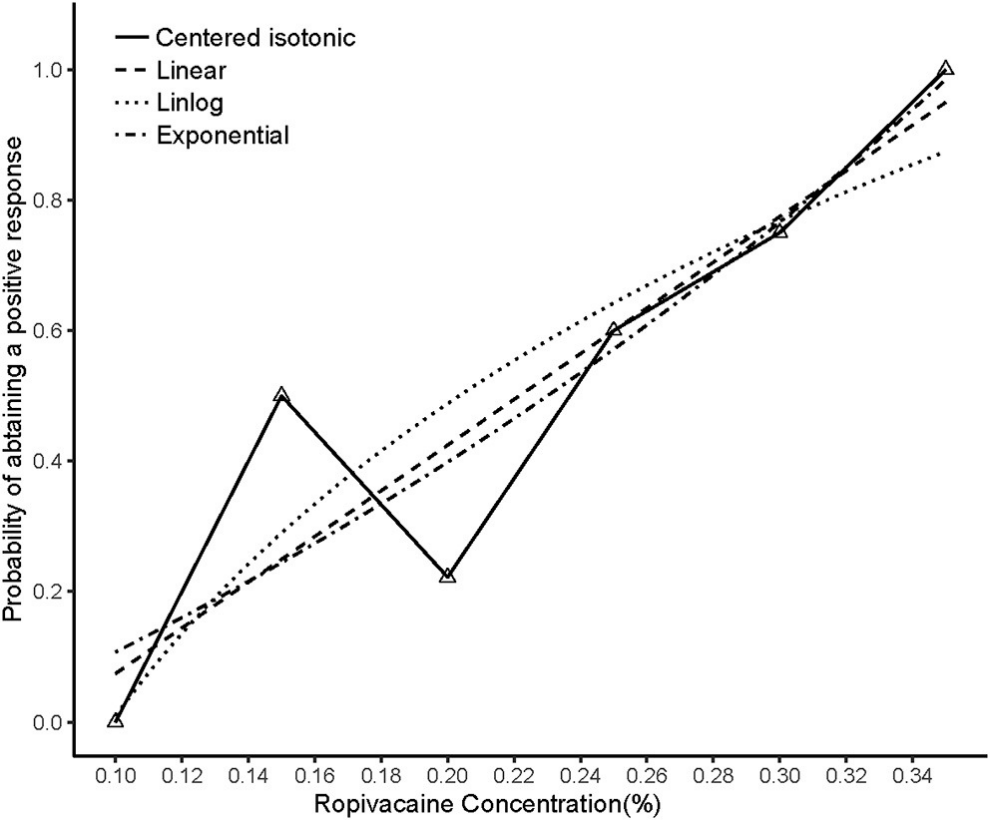
Estimated ropivacaine–Interscalene Brachial Plexus Block relationship for a given dose level and probability of successful block. Median estimators for each model are plotted. The numbers of measurements at each ropivacaine concentration are represented by numbered triangles.

The illustration of the sequence of successful and unsuccessful postoperative analgesia is shown in Table 2. The linear model estimator led to an EC50 of 0.222%, the linear-logarithmic model resulted in an EC50 value of 0.233%, the exponential regression gave an EC50 of 0.223%, and the centered isotonic regression (a nonparametric method) yielded an EC50 of 0.232% (see Figure 2). The 95% confidence intervals for the 3 parametric models (linear, linear-logarithmic, and exponential) were 0.198%, 0.335%; 0.215%, 0.453%; and 0.202%, 0.436%, respectively (Table 2), and they showed similar fitted probabilities within the range of the EC50, while the 95% confidence intervals from these models successfully covered all observed data. Table 2 also shows the results of residual standard deviations for the goodness of fit of each model. The exponential regression has the least residual standard error (0.1676) among all models.

**Table 2:** The mean effective concentration and 95% confidence interval of the different models.

**Model**	**ED 50 (%)**	**95%CI(%)**	**Residual standard error**
**Centered isotonic**			
Regression	0.232		
Linear	0.222	0.198, 0.335	0.1676
Linlog	0.233	0.215, 0.453	0.1823
Exponential	0.223	0.202, 0.436	0.1907

### Block Performance Characteristics

The mean onset time for the sensory block to reach grade 1 was 5.8 ± 3.33 min and the mean onset time for the motor block to reach grade 2 was 12.9 ± 2.81 min. The onset time of sensory block and motor block was not significantly different between patients having successful and failed blocks (*p* = 0.5890, *p* = 0.7012, respectively). All patients achieved grade 1 or 2 with motor block within 8 h after surgery. The average duration of the motor block was 7.5 ± 1.32 h. No difference occurred in the duration of the motor block between successful and unsuccessful blocks (*p* = 0.6500).

### Postoperative Pain and Rescue Analgesia Required

**Figure 3:**
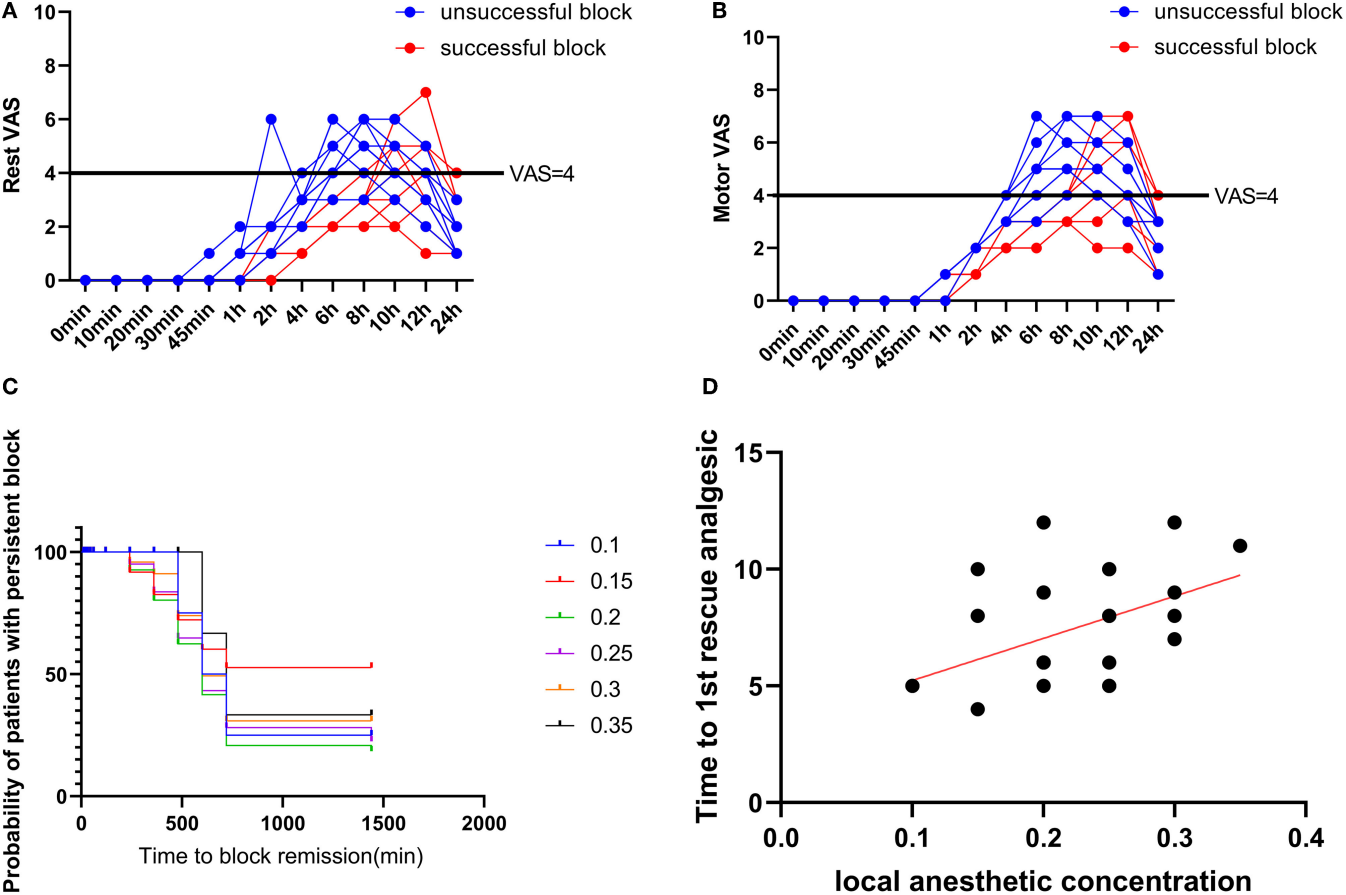
Postoperative pain scores. **(A)** Rest pain score 24 h after surgery. **(B)** Motor pain score 24 h after surgery. **(C)** Duration of the Interscalene Brachial Plexus Block with different concentrations of ropivacaine. **(D)** Correlation between ropivacaine concentration and time to first rescue analgesic in interscalene brachial plexus block.

Out of the total patients included in the study, 16 patients had a successful block. All patients with a successful block had a postoperative visual analog scale score of < 4 in the initial 8 h (Figures 3A,B). The average intraoperative sufentanil consumption was 10.8 ± 3.33 μg. Intraoperative sufentanil consumption between successful and unsuccessful blocks (*p* = 0.6676) showed no difference. However, the mean time to first rescue analgesia was 9.2 ± 2.71 h. The time to 1st rescue analgesia between successful and unsuccessful blocks (*p* < 0.0001) was significantly different. The time to 1st analgesic request was moderately positively correlated with administered local anesthetic concentration, with the Spearman rank correlation (r) being 0.4351. This value of r was found to be statistically significant (*p* = 0.0163) (Figures 3C,D).

### Postoperative Adverse Events

A female patient complained of chest tightness on the blocked side after returning to the ward, suggesting phrenic nerve block and unilateral lung function decline. This was relieved by nasal cannula oxygen inhalation, without hypoxemia occurrence. No other complications were noted.

## Discussion

In this study, we have found the median EC50 was 0.222% (95% CI, 0.202 to 0.436%).

The ISBPB can provide dense analgesia and anesthesia to the upper extremity from the shoulder to the fingers, depending on the indication and approach utilized. The use of ultrasound has made the block more accessible and safer to perform. There is evidence to suggest that the use of ultrasound reduces the total volume of anesthetic required, decreases complications such as pneumothorax and vascular injury, and increases block success (13). Therefore, general anesthesia combined with ultrasound-guided nerve block is the preferred method compared to general anesthesia alone, particularly when general anesthesia with a laryngeal mask that preserves the patient's spontaneous breathing (14). Compared with endotracheal intubation, it can reduce or circumvent irritation to the soft tissues of the pharynx and tracheal wall, and improve the hemodynamic stability of anesthesia induction and recovery period. Meanwhile, the amount of medicine required by the laryngeal mask has also been reduced in contrast to the endotracheal intubation. Compared with simple intravenous anesthesia, considering the special “beach chair position” or “semi-sitting position,” sedative analgesics can be used more safely under the premise of a laryngeal mask, which improves the safety of airway and patient comfort.

With regard to proximal humerus fracture operation, ISBPB is effective in postoperative pain control and reducing opiate intraoperative use in patients. Various approaches can be considered, such as a suprascapular nerve block (SSNB) or a superior trunk block (15, 16). Several randomized controlled trials have compared ISBPB with SSNB, but the evidence is conflicting. Some have found ISBPB to be superior, whereas others have shown that SSNB provides non-inferior analgesia (17). A review suggested that there are no clinically meaningful analgesic differences between ISBPB and SSNB except that ISBPB does provide better pain control during recovery room stay (18). The superior trunk block can potentially cause diaphragm sparing, but further research is needed to determine the efficacy (16). Thus, ISBPB is the most popular and frequently used approach for proximal humerus fracture operation.

Ropivacaine is one of the commonly used drugs for nerve block. It has the characteristics of motor-sensory block separation at low concentrations meaning the sensory function of the corresponding body parts is temporarily lost, while the motor function can be partially or completely retained. Studies have found that brachial plexus block using 0.10–0.25% Ropivacaine can achieve the separation of sensory and motor (9). Patients undergoing proximal humerus fracture operation are required for early functional exercises. Therefore, a brachial plexus block with a low concentration of Ropivacaine is an ideal method of anesthesia and postoperative analgesia.

When performing ISBPB, there is a high risk of causing ipsilateral hemidiaphragmatic paralysis *via* phrenic nerve palsy (19). For patients without basic respiratory diseases before surgery, even if diaphragmatic paralysis occurs, the postoperative respiratory function of patients can still be well-tolerated (20). Therefore, none of the patients enrolled in this study had preoperative pulmonary disorders. A large number of studies have shown that the incidence of phrenic nerve block is 100% when the volume of Ropivacaine used in ISBPB exceeds 15 ml (21). Meanwhile, It has been reported that when 0.75% Ropivacaine is used for ISBPB, an average of 1.7 ml of local anesthetic for each nerve root can meet the needs of a single nerve block (22). Therefore, in this study, due to the expected low target concentration of Ropivacaine, a total volume of 10 ml LA was used to block the brachial plexus. To achieve a more satisfactory blocking effect, 3–4 ml drug was injected around the three roots, respectively, and all blocks were completed under ultrasound guidance to ensure the accuracy of the injection site. Previously, it has been reported that the EC50 of surgical operation under nerve block using Ropivacaine alone is 0.2675% (23). Thus, an initial concentration of 0.3% for ISBPB was selected.

The Dixon and Mood up-and-down sequential method is used to assess the dose-response of medications. It proved to be an effective method with reduced samples compared to classic studies of multiple groups with fixed concentrations. In this study, the linear model was used to calculate the EC50 of Ropivacaine for postoperative analgesia of proximal humerus fracture after general anesthesia combined with ISBPB. The EC50 measured by other methods is not much different from this result and is less than commonly used clinical doses. Therefore, during general anesthesia combined with a nerve block, the concentration of Ropivacaine can be appropriately reduced.

Also, this study has certain limitations, although we strictly abide by the entry standards, follow the operating specifications, and conduct the experiments by the blind method. There may be selection bias due to the small sample size in the study; thus, the experimental results still need to be further verified by large samples and multi-center studies. In addition, a VAS score < 4 points within 8 h after the operation was defined as a standard for a successful block in this study; otherwise, it is recognized as unsuccessful. The VAS score test is highly subjective and may affect the experimental results.

In conclusion, we found that the median EC50 of Ropivacaine is 0.222%.

## Data Availability Statement

The original contributions presented in the study are included in the article/supplementary material, further inquiries can be directed to the corresponding author/s.

## Ethics Statement

The studies involving human participants were reviewed and approved by Ethics Committee of the Sixth People's Hospital of Shanghai. The patients/participants provided their written informed consent to participate in this study.

## Author Contributions

CX and YL wrote the manuscript. JL designed the research. CX, FG, RC, and CW performed the research. CX and CW analyzed the data. FG and RC contributed new reagents and analytical tools. All authors agree to the submission of this manuscript.

## Conflict of Interest

The authors declare that the research was conducted in the absence of any commercial or financial relationships that could be construed as a potential conflict of interest.

## Publisher's Note

All claims expressed in this article are solely those of the authors and do not necessarily represent those of their affiliated organizations, or those of the publisher, the editors and the reviewers. Any product that may be evaluated in this article, or claim that may be made by its manufacturer, is not guaranteed or endorsed by the publisher.
